# Solidified Floating Organic Drop Microextraction for the Detection of Trace Amount of Lead in Various Samples by Electrothermal Atomic Absorption Spectrometry

**DOI:** 10.1155/2017/6268975

**Published:** 2017-07-20

**Authors:** Oya Aydın Urucu, Şeyda Dönmez, Ece Kök Yetimoğlu

**Affiliations:** Department of Chemistry, Marmara University, Göztepe, 34722 Istanbul, Turkey

## Abstract

A novel method was developed for determination of trace amounts of lead in water and food samples. Solidified floating organic drop microextraction was used to preconcentrate the lead ion. After the analyte was complexed with 1-(2-pyridylazo)-2-naphthol, undecanol and acetonitrile were added as extraction and dispersive solvent, respectively. Variables such as pH, volumes of extraction and dispersive solvents, and concentration of chelating agent were optimized. Under the optimum conditions, the detection limit of Pb (II) was determined as 0.042 *µ*g L^−1^ with an enrichment factor of 300. The relative standard deviation is <10%. Accuracy of the developed procedure was evaluated by the analysis of certified reference material of human hair (NCS DC 73347) and wastewater (SPS-WW2) with satisfactory results. The developed procedure was then successfully applied to biscuit and water samples for detection of Pb (II) ions.

## 1. Introduction

Lead is an important naturally occurring heavy metal. It is found in small amounts in the earth's crust and is used in many areas including industrial, agricultural, and domestic areas [1, 2]. Lead is used in many industrial processes such as metal products, lead-acid batteries, manufacturing of lead based paints, and automobile exhaust accumulators, ammunitions, and so on. Humans can be exposed to lead by air, water, or food contamination. This creates serious health risks for both humans and animals. Lead accumulates in soft tissues in young and middle-aged people and in bones in older people. The accumulation of lead in the kidneys and liver is also quite high [1, 3]. In the liver, tetramethyl lead turns into a much more toxic triethyl lead and causes the increase in the amount of lead in the urine. The World Health Organization (WHO) recommended a limit of 10 *µ*gL^−1^ of lead in potable water [4] and according to International/National Standards, maximum permitted concentration of lead in all food in solid form is 6 *µ*gL^−1^.

The detection of heavy metals in various samples is important for human health. The most commonly used instrumental methods are flame atomic absorption spectrometry (FAAS), electrothermal atomic absorption spectrometry (ETAAS), inductively coupled plasma optical emission spectrometry (ICP-OES), and inductively coupled plasma-mass spectrometry (ICP-MS) [5]. Direct detection of trace amounts of heavy metal ions in real samples by AAS is difficult due to matrix effects and a low detection limit is difficult [6]. The easiest way to deal with this difficulty is to use separation-preconcentration method before analysis. In recent years, a number of easily applicable separation-preconcentration methods have been developed. These methods require the use of a small amount of organic solution. The most commonly used methods are single drop microextraction (SDME) [7, 8], coprecipitation [9], solid phase microextraction (SPME) [10, 11], cloud point extraction (CPE) [12], dispersive liquid-liquid microextraction (DLLME) [13, 14], and solidified floating organic drop microextraction (SFODME) [15, 16].

**Figure 1:**
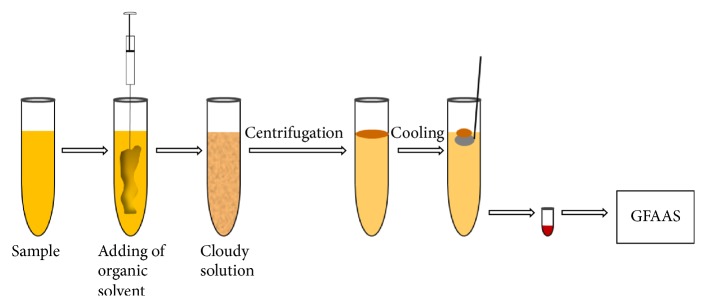
Schematic diagram of DLLME-SFO method.

The most important feature of the SFODME method is that the analyte in the aqueous sample is trapped in a small drop of the extract solution. The density of organic solvents used in this method is less than the density of water. In SFODME method, a mixture of dispersive and extraction solvents is quickly injected into aqueous sample by microsyringe and the solution becomes cloudy. Then the extraction solution is separated by centrifuging the cloudy solution. The test tube is placed in the ice bath for the freezing of the organic phase. Then the extraction solution is collected in another test tube using a spatula. The organic droplet melts at room temperature and is dissolved in a suitable solvent before analysis is ready. A schematic diagram of SFODME is shown in Figure 1. This method is a widely accepted, optimal, and powerful technique because of its use of negligible volumes of solvents, high enrichment factor, low cost, low consumption of organic solvents, simplicity of automation, ability to detect analytes at very low concentrations, and the ability to be coupled with various modern detection techniques [24, 25].

In this study, we selected 1-(2-pyridylazo)-2-naphthol (PAN), which is the classical organic reagent for spectrophotometric determination of transition metal ions as a chelating reagent. We have developed a new SFOMDE method combined with GFAAS for detection and separation of lead in various samples. We have optimized the factors affecting the extraction efficiency such as pH, sample volume, ionic strength, concentration of organic ligands, and solvent volume.

## 2. Experimental

### 2.1. Instrumentation

An AAS ZEEnit 700P atomic absorption spectrometer (Analytik Jena AG, Jena, Germany) with deuterium background correction, equipped with a transversely heated graphite tube atomizer, was used for all measurements. The temperature program for the ETAAS is shown in Table 1. The lead hollow cathode lamp (Analytik Jena AG, Konrad, Zuse, Germany) was operated at 4.0 mA and the analytical line at 283.3 nm was used with a spectral bandwidth of 0.5 nm. Mettler Toledo pH meter was used to measure the pH. An analytical Precisa XB 220A balance was used for all weights measurements and Hettich EBA 21 model centrifuge was used for centrifugation. A CEM MARS microwave digestion system was used for ore sample digestion.

### 2.2. Reagents and Standard Solutions

In this study, the reagents of the highest purity and analytical grade were used. The standard stock solution of Pb (II) (1000 mgL^−1^) was prepared by dissolving 1,599 g of Pb(NO_3_)_2_ (Merck) in 1000 mL of 0.5% nitric acid solution. The working solutions of the Pb(II) ions were prepared by suitable dilution from stock solution using deionised water. 1-Undecanol was used as an extracting solvent (Merck), while acetonitrile was used as dispersive solvent.

1-(2-Pyridylazo)-2-naphthol (PAN) solution was prepared by dissolving 0.01246 g of PAN (Sigma-Aldrich) in of 50 ml of ethanol. NH_3_/NH_4_Cl buffer solutions were prepared by mixing appropriate amounts of 1.0 mol L^−1^ NH_3_ (Merck) and 1.0 mol L^−1^ NH_4_Cl (Merck) solutions for pH values from 8 to 10.5.

### 2.3. Microwave Digestion of Certified Reference Material (CRM)

50 mg CRM (NCS DC 73347) weights were transferred into the microwave vessels and then 6 mL HNO_3_ (65% w/v), 3 mL H_2_O_2_ (30% w/v), and 1 mL HF were added. Optimized microwave program (180°C for 20 min) was applied. This vessel was cooled to room temperature and then the volume was brought up to 25 mL with distilled water.

### 2.4. General Procedure

Aliquots of 10 mL standard solution containing 10 *μ*gL^−1^ Pb were placed in a 15 mL conical centrifuge tube. The pH was adjusted to 9 with a NH_3_/NH_4_Cl buffer, while ionic strength was set to 20% by using NaCl. The amount of 1 ml PAN (1 × 10^−4^ mol L^−1^), 100 *µ*L 1-undecanol (extraction solvent), and 200 *µ*L acetonitrile (dispersive solvent) was quickly injected into the sample solution by using a syringe. A cloudy mixture was formed in the sample tube indicating that Pb reacted with PAN. The mixture was centrifuged for 15 min at 5000 rpm, allowing the separation of the organic phase at the top of the tube. After cooling the sample tube in an ice bath, the organic phase was transferred into another tube and diluted to 500 *μ*L with ethanol. The solution was then analyzed by using the GFAAS.

## 3. Result and Discussion

The effects of different experimental parameters such as the extraction time, concentration of chelating agent and complex with standard solution, buffer solution selection, pH, extraction volume, dispersive solvent, salt addition, and sample volume were investigated to obtain the optimum SFODME conditions.

### 3.1. Effect of Type and Volume of Extraction Solvent

**Figure 2:**
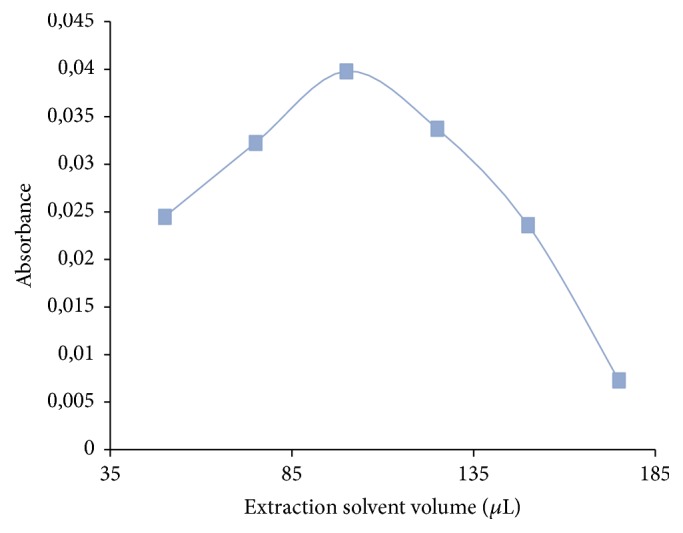
The effect of the extraction solvent volume on absorbance of lead with SFODME. Extraction circumstances: Pb: 10 *µ*gL^−1^; pH: 9; extraction solvent, 1-undecanol (100 *µ*L); sample volume, 10 mL; dispersive solvent, acetonitrile (200 *µ*L); % 1.6 NaCl; chelating reagent [PAN], 1 × 10^−4^ mol L^−1^.

The selection of a correct extraction solvent is crucial for optimization of the SFODME process. Extraction solvent must have low volatility, solubility, and toxicity, be stable for the duration of extraction, extract required analytes well, and have a melting point close to the room temperature. Based on this information, we tested two different organic extraction solvents such as 1-undecanol and 1-dodecanol. The experimental results demonstrated that the best extraction of the target analyte was achieved when 1-undecanol was used as the extraction solvent. Hence, solutions including different volumes of 1-undecanol (50–175 *µ*l) were applied to the same SFODME procedure to investigate the effect of solvent volume on extraction. The results are shown in Figure 2.

According to the results, the extraction recovery increased with the increased volume of the extraction solvent; however, the recovery was maximum when volumes of more than 100 *µ*l were used. Therefore, 100 *µ*l of 1-undecanol was decided as the optimum volume of extraction solvent to achieve good sensitivity and high enrichment.

### 3.2. Effect of Type and Volume of Dispersive Solvent

**Figure 3:**
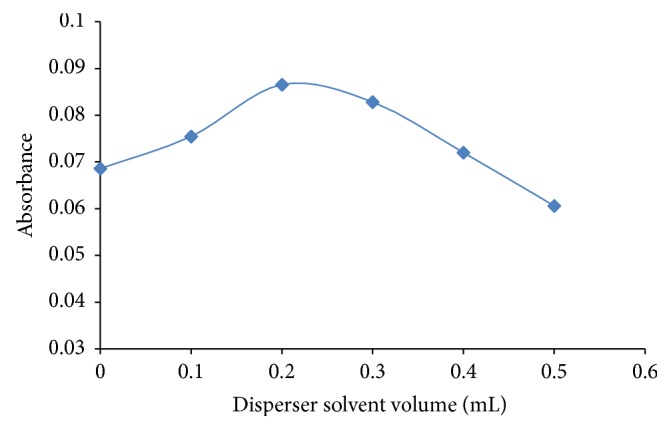
The effect of dispersive solvent volume on absorbance of lead for SFODME. Extraction circumstances: Pb: 10 *µ*gL^−1^; pH: 9; extraction solvent, 1-undecanol (100 *µ*L); sample volume, 10 mL; pH: 9; % 1.6 NaCl chelating reagent [PAN], 1 × 10^−4^ mol L^−1^.

In order to be successfully used in SFODME, the selected dispersive solvent must be miscible with both water and extraction solvents. A cloudy solution should be obtained when dispersive solvent is injected together with the extraction solvent and ligand into aqueous mixture. Dispersive solvent must have a low cost, low toxicity, and low volatility. Various dispersive solvents such as methanol, acetone, ethanol, and acetonitrile were tested. The recovery was the highest with acetonitrile; therefore, acetonitrile was selected as dispersive solvent. Subsequently, the volume of acetonitrile was optimized by testing the 100–500 *µ*L range in terms of the extraction efficiency (the volume of 1-undecanol as extraction solvent was added at 100 *µ*L). As shown in Figure 3, the extraction efficiency increased with increasing volume of acetonitrile. The highest recovery was obtained with 200 *µ*L. Hence, 200 *µ*L was selected as the optimum volume of the dispersive solvent.

### 3.3. Effect of Sample Solution pH

**Figure 4:**
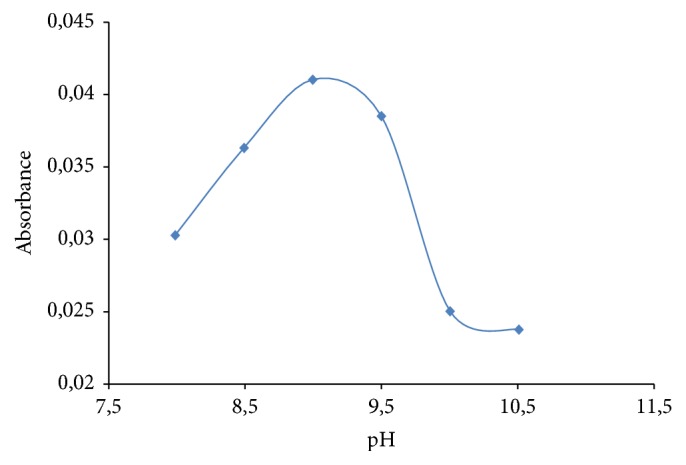
The effect of pH on the extraction of lead with SFODME. Extraction circumstances: Pb: 10 *µ*gL^−1^; extraction solvent, 1-undecanol (100 *µ*L); sample volume, 10 mL; dispersive solvent, acetonitrile (200 *µ*L); % 1.6 NaCl; chelating reagent [PAN], 1 × 10^−4^ mol L^−1^.

pH is one of the main parameters necessary for the ligand-metal complex reaction. pH between 9.0 and 10.0 is favorable for complexation of lead and PAN [26]. Therefore, the effect of pH on the SFODME extraction of lead was investigated in the pH range of 8–10.5 by using NH_3_/NH_4_Cl buffer solution, while keeping all other factors constant. As shown in Figure 4, the extraction recovery was almost constant at pH bigger than 9. Therefore, pH 9 that was adjusted with NH_3_/NH_4_Cl buffer solution was accepted as the optimum pH value and all subsequent sample analyses were done at pH 9.

### 3.4. Effect of the Concentration of 1-(2-Pyridylazo)-2-naphthol

**Figure 5:**
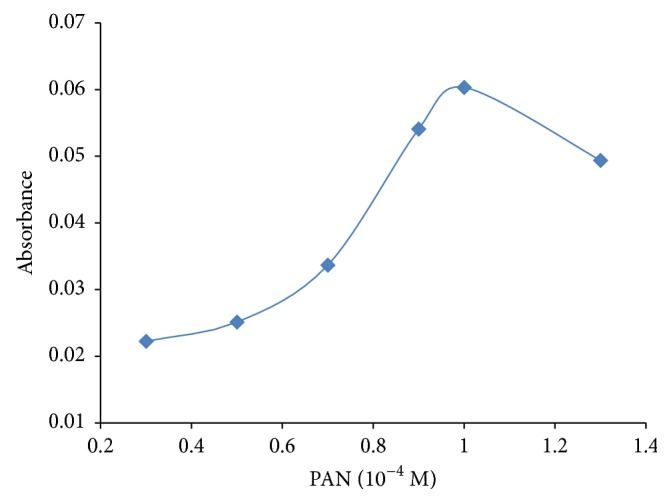
The effect of 1-(2-pyridylazo)-2-naphthol concentration on absorbance of lead with SFODME. Extraction circumstances: Pb: 10 *µ*gL^−1^; pH: 9; extraction solvent, 1-undecanol (100 *µ*L); sample volume, 10 mL; dispersive solvent, acetonitrile (200 *µ*L); chelating reagent [PAN], 1 × 10^−4^ mol L^−1^.

1-(2-Pyridylazo)-2-naphthol (PAN) is a classical spectrometric reagent used for analysis of metal ions. The metal chelates of PAN have the metal atom bonded to O of the OH group, to pyridine N, and to azo N [27]. In this study, PAN formed a strong complex with the Pb ions at pH 9. The effects of various PAN concentrations ranging from 0.2 × 10^−4^ to 1.4 × 10^−4^ mol L^−1^ on extraction efficiency were investigated. The results are demonstrated in Figure 5.

According to these results, increasing concentrations of PAN resulted in higher recovery but it was stable at 1 × 10^−4^ mol L^−1^. For this reason, 1 × 10^−4^ mol L^−1^ was chosen as optimum concentration for this study.

### 3.5. Effect of Salt Addition

In this study, the effect of ionic strength on the SFODME performance was tested with NaCl concentration ranging from 0 to 4%, while all other parameters were constant. Salt addition increased the extraction efficiency but the efficiency reached the plateau at 1.6% NaCl concentrations. Thus, the optimal salt amount was determined as 1.6% NaCl.

### 3.6. Effect of Different Ions

The effect of potential interfering cations was tested in optimal conditions in order to measure the degree of usefulness of newly developed SFODME method in analytical applications. Hence, according to the detected procedure, 10.0 ml of solutions containing 10 *µ*gL^−1^ of lead and varied quantity of interfering ions were treated. The results are shown in Table 2. Based on the results shown in Table 2, specified amount of cations have no effect on the quantitative determination of the amount of lead in the sample.

### 3.7. Effect of Sample Volume

The effects of sample volume on the recovery of Pb (II) ions were investigated with volumes ranging from 10 mL to 100 mL by using model solutions prepared at the optimal conditions. Quantitative recovery was obtained for every volume. The preconcentration factor for Pb(II) calculated by taking the ratio of the highest sample volume (100 mL) and the lowest final volume (0,3) was 333, while the enhancement factor, which is the ratio of the slope of calibration curve of the analytes after preconcentration to that prior to the preconcentration, was 300.

### 3.8. Analytical Performance of the Procedure

The linear range of SFODME procedure was calculated by analyzing different Pb(II) ion concentrations. The linear dynamic range was determined as 0.05–40 *µ*gL^−1^ with the correlation coefficient (*R*^2^) exceeding 0.9986. The detection limit, which is defined as the concentration equivalent to three times the standard deviation (*N* = 10) of the reagent blank, was 0.042 *µ*gL^−1^. The recovery values for the analytes were between 91 and 100 percent. Meanwhile, the relative standard deviation (RSD%) was determined to be <10%.

### 3.9. Applications of the Presented Method

The proposed SFODME procedure was applied to detect Pb (II) ion in different samples. Firstly, the accuracy of the method was verified by using NCS DC 73347 (hair reference material) and SPS-WW2 (wastewater), which are certified reference material (Table 3). Prior to the analysis, hair reference material was digested by close microwave. The amount of Pb (II) ion in tap and river water samples was detected without any pretreatment to the samples (Table 4). The proposed method was also applied to the food samples. The contents of the food sample (biscuit) have also been detected with other methods (Table 3).

## 4. Conclusions

A novel SFODME method was developed for the preconcentration of Pb (II) ions in hair, food, and water samples. This method is a fast, simple, sensitive, and accurate technique for the preconcentration and detection of Pb (II). The different ions effect was tolerable. The limit of detection and enrichment factor found in this study were superior to other preconcentration techniques in terms of detection of lead (Table 5). The present method has high enrichment factor and low detection limit; thus, it is suitable for ultratrace analysis of lead in different types of samples. In addition, our method uses very little organic solvent and therefore is environment-friendly.

## Figures and Tables

**Table 1:** The temperature program of graphite furnace atomizer.

Step	Start *T* (°C)	End *T* (°C)	Ramp time (s)	Hold time (s)	Ar flow rate (mL min^−1^)
Drying	80	140	40	2	250
Ashing	500	500	20	2	250
Atomizing	1400	1400	0	5	0
Clean	2200	2200	0	4	250

**Table 2:** Effect of the coexisting ions on the recoveries of the Pb (II).

Coexisting ions	Molar ratio (ion/Pb^+2^)
Ba^+2^	2000
K^+^	2000
Cd^+2^	2000
Ni^+2^	800
Cu^+2^	800
Ag^+^	200
Zn^+2^	200
Mg^+2^	200
Hg^+2^	200
Co^+2^	200
Fe^+3^	200

**Table 3:** Determination of Pb (II) ions in human hair and biscuit samples.

Sample	Certified Pb (II) (*µ*g L^−1^)	Found (*µ*g L^−1^)	Recovery (%)
Hair reference material (NCS DC 73347)	8,8	8,5 ± 0.02	96.6 ± 2
Biscuit	24	24.10 ± 0.05	100 ± 1
Wastewater (SPS-WW2)	500	495	99

**Table 4:** Determination of Pb (II) ions in tap-water and river-water samples.

Sample	Added (Pb^2+^) (*µ*g L^−1^)	Found (*µ*g L^−1^)	Recovery (%)
Tap water	0	—	—
	12.5	12.32 ± 0.03	98.56 ± 2
	15	14.77 ± 0.02	98.46 ± 2
	17.5	16.0 ± 0.01	91.42 ± 1

River water	0	—	—
	12.5	11.98 ± 0.01	95.84 ± 1
	15	14.74 ± 0.02	98.26 ± 2
	17.5	16.66 ± 0.01	95.20 ± 1

**Table 5:** Comparison of the SFODM method with some recent studies on separation and preconcentration of Pb(II) ions reported in the literature.

Technique	Sample	E.F	LOD (*µ*gL^−1^)	Liner range (*µ*gL^−1^)	R
CPE-FAAS	Water and food samples	25	3.42	500–10000	[17]
Coprecipitation-FAAS	Water samples	40	9,7	—	[18]
HF-LPME-ETAAS	Environmental and biological samples	76	0.02	0,04–1	[19]
DLPME-GFAAS	Water and biological samples	78	0.039	0.004–0.03	[2]
SPE-FAAS	Environmental water and vegetable samples	125	0.61	10–600	[20]
SM-DLLME-FAAS	Food samples	—	0.4	1–500	[21]
SPE-FAAS	Water samples	20	5	1–12	[22]
SFODME-GFAAS	Water and rack samples	113	0,058	0,2–10	[23]
SFODME-GFAAS	Water and food samples	300	0.042	0.05–40	This study

CPE: cloud point extraction; DLPME: dispersive liquid phase microextraction; HF-LPME: hollow fiber based-liquid phase microextraction; SPE: solid phase extraction; SFODME: solidified floating organic drop microextraction; ETAAS: electrothermal atomic absorption spectrometry; GFAAS: graphite furnace atomic absorption spectrometry; FAAS: flame atomic absorption spectrometry; LOD: limit of detection; E.F: enrichment factor; SM: supramolecular microextraction; R: reference.
